# Sequential occurrence of eclampsia-associated posterior reversible encephalopathy syndrome and reversible splenial lesion syndrome (a case report): proposal of a novel pathogenesis for reversible splenial lesion syndrome

**DOI:** 10.1186/s12880-019-0323-7

**Published:** 2019-04-30

**Authors:** Qing Yang, Can-can Chang, Mengxiao Liu, Yong-qiang Yu

**Affiliations:** 1 Department of MRI, Anqing Hospital Affiliated to Anhui Medical University(Anqing Municipal Hospital), Anqing, 246000 Anhui China; 2 Department of Medical Imaging, Huatuo Hospital of Traditional Chinese Medicine, Bozhou, 236800 Anhui China; 3 Scientific Marketing, Siemens Healthcare, Shanghai, China; 4 The First Affiliated Hospital of Anhui Medical University, Meishan road, Hefei, 230032 Anhui China

**Keywords:** Eclampsia, Posterior reversible encephalopathy syndrome, Reversible splenial lesion syndrome, Magnetic resonance imaging

## Abstract

**Background:**

Posterior reversible encephalopathy syndrome (PRES) is a rare clinic-radiological entity characterized by headache, an altered mental status, visual disturbances, and seizures. Reversible splenial lesion syndrome (RESLES) is a new clinic-radiological syndrome characterized by the presence of reversible lesions with transiently restricted diffusion (cytotoxic edema) in the splenium of the corpus callosum (SCC) on magnetic resonance (MR) images. Here we report a rare case involving a 23-year-old pregnant woman with eclampsia who sequentially developed PRES and RESLES.

**Case presentation:**

The patient, a 23-year-old pregnant woman, presented with sudden-onset headache, dizziness, and severe hypertension (blood pressure, 170/110 mmHg). Brain MR imaging (MRI) revealed T2 hyperintense lesions in the posterior circulation territories. Immediate cesarean section was performed, and the patient received intravenous infusion of mannitol (125 ml, q8h) for 8 days for the treatment of PRES. Ten days later, or 1 day after the discontinuation of mannitol, T2-weighted MRI showed that the hyperintense lesions (vasogenic edema) had disappeared. However, diffusion-weighted imaging (DWI) and apparent diffusion coefficient (ADC) mapping revealed an isolated lesion in the splenium of the corpus callosum (SCC) that was accompanied by restricted diffusion (cytotoxic edema); these findings indicated reversible splenial lesion syndrome (RESLES). Five days after the discontinuation of mannitol, she had no abnormal symptoms and was discharged from our hospital. Brain MRI performed 29 days after the clinical onset of symptoms showed no abnormalities.

**Conclusion:**

The sequential occurrence of the two reversible diseases in our patient prompted us to propose a novel pathogenesis for RESLES. Specifically, we believe that the vasogenic edema in PRES was reduced with mannitol treatment, which increased the hyperosmotic stress and opened the blood–brain barrier; meanwhile, upregulation of aquaporin-4 expression secondary to the increased osmotic pressure resulted in cytotoxic edema in the astrocytes in SCC (RESLES). Further research is necessary to confirm this possible pathogenesis.

## Background

Posterior reversible encephalopathy syndrome (PRES) is a rare clinico-radiological entity characterized by headache, an altered mental status, visual disturbances, and seizures [1]. Characteristic radiological findings include symmetric vasogenic edema [mild hyperintensity in diffusion-weighted imaging (DWI) and apparent diffusion coefficient (ADC) mapping] in the posterior cerebral regions, particularly the parietooccipital lobes [2]. The prognosis is usually benign, with complete reversal of clinical symptoms within several days, when adequate treatment is immediately initiated. Reversible splenial lesion syndrome (RESLES) is a new clinico-radiological syndrome characterized by the presence of reversible lesions with transiently restricted diffusion (cytotoxic edema) in the splenium of the corpus callosum (SCC) on magnetic resonance (MR) images. The syndrome was once named: Clinically mild encephalitis/encephalopathy with a reversible splenial lesion (MERS). Clinically, this syndrome is associated with a wide spectrum of diseases or a variety of factors, including encephalopathy, seizures, antiepileptic drug withdrawal, and the use of anticancer drugs [3]. The neurological symptoms of RESLES include delirious behavior, a short-term disturbance in consciousness, and seizures, and most patients exhibit complete recovery without neurological sequelae after a short disease course [4]. However, the underlying pathophysiological mechanism remains unknown. There are scant, if any, cases described of concomitant PRES- RESLES(MERS), although preliminary cases of acute toxic leukoencephalopathy (ATL) have been described with PRES [5]. Here we report a rare case involving a 23-year-old pregnant woman with eclampsia who sequentially developed PRES and RESLES.

## Case presentation

**Fig. 1:**
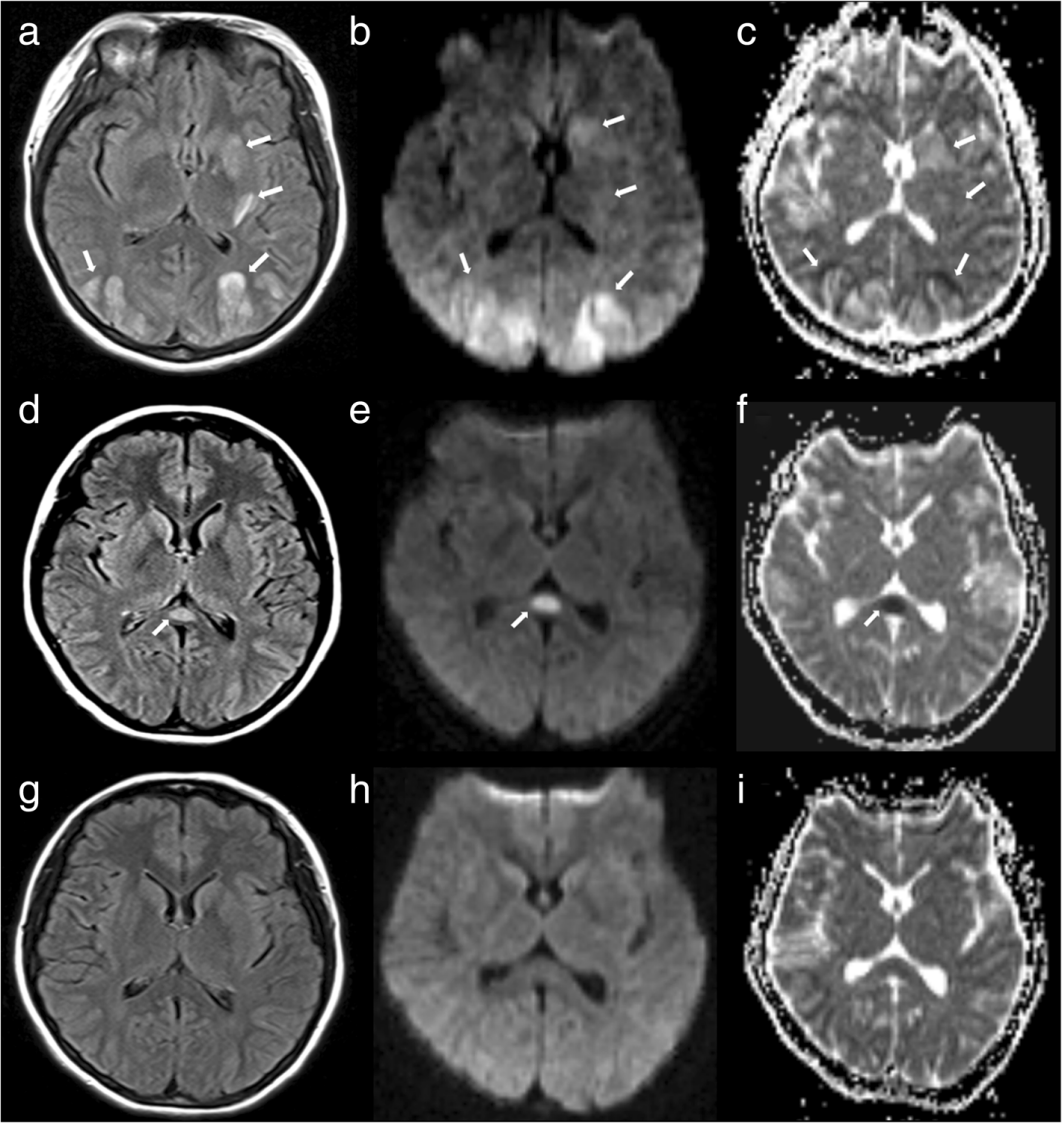
Brain magnetic resonance (MR) images for a 23-year-old woman with eclampsia who presented with sudden-onset headache, dizziness, and severe hypertension (blood pressure, 170/110 mmHg). **a**–**c**. Axial brain MR images obtained at the clinical onset of symptoms show vasogenic cerebral edema in the bilateral parietooccipital lobes, left basal ganglia, and corona radiata. Fluid-attenuated inversion recovery (FLAIR) imaging (**a**), diffusion-weighted imaging (DWI; b = 1000; **b**), and apparent diffusion coefficient (ADC) mapping (**c**) show mild hyperintensity in the lesions (arrows), which is a typical finding in posterior reversible encephalopathy syndrome (PRES). **d**–**f**. Follow-up MR images obtained 10 days after the clinical onset of symptoms (i.e., 1 day after withdrawal of mannitol show that the originally observed hyperintense lesions have disappeared (vasogenic edema has disappeared). FLAIR imaging (**d**) shows an isolated hyperintense signal in the splenium, whereas DWI (**e**) and ADC mapping (**f**) show restricted diffusion in the splenium (arrows), which is a typical finding in reversible splenial lesion syndrome. g–i. Follow-up MR images obtained 29 days after the clinical onset of symptoms FLAIR imaging (**g**), DWI (**h**), and ADC mapping (i) show that the lesions in the splenium have disappeared

A 23-year-old pregnant woman without any history of hypertension or migraine suddenly developed a thunderclap headache, dizziness, and eye pain at 35 + 2 weeks of gestation. She did not take these symptoms seriously and also experienced one episode of vomiting without fever and syncope. By the afternoon of the same day, her symptoms worsened, and she was admitted to the emergency department with a complaint of mistiness of vision in both eyes. At the time of admission, her blood pressure was 170/110 mmHg. Neurological examination revealed no abnormal signs such as hemiparesis and seizures. Serological laboratory tests showed no autoimmune conditions or infectious pathogens such as bacteria and viruses. Brain MRI performed on the same day revealed symmetric lesions in the posterior circulation territories, including the bilateral parietooccipital lobes, left basal ganglia, and corona radiata. These lesions showed hyperintensity on T2-weighted imaging and fluid-attenuated inversion recovery (FLAIR) imaging (Fig. 1a). DWI (Fig. 1b) and ADC mapping (Fig. 1c) revealed mild hyperintensity in the lesions, which indicated vasogenic cerebral edema. The patient was diagnosed with eclampsia-associated PRES and received intravenous infusion of mannitol (125 ml; q8h × 8 days) for the management of intracranial hypertension. On the same day, cesarean section was successfully performed, and her blood pressure decreased to 154/103 mmHg one hour after surgery. However, she complained of headache and bilateral blindness. On the day after surgery, her headache ameliorated, vision improved, and blood pressure decreased to 140/85 mmHg. Ten days later, i.e., one day after the withdrawal of mannitol, FLAIR imaging, DWI, and ADC mapping showed that the hyperintense lesions (vasogenic edema) had disappeared. However, an isolated lesion with restricted diffusion that showed a high signal in DWI and a low ADC value (cytotoxic edema) was observed in SCC; these findings indicated RESLES type I) [6]. Five days after the discontinuation of mannitol, she showed no abnormal symptoms and was discharged from our hospital. Follow-up brain MRI performed 29 days the clinical onset of symptoms showed no abnormalities (Fig. 1g–i).

## Discussion

In this report, we described the sequential occurrence of eclampsia-associated PRES and RESLES in a young woman. To the best of our knowledge, this phenomenon has not been previously reported. The case presented special MRI findings wherein vasogenic edema (PRES) disappeared and cytotoxic edema (RESLES) developed because of reasons unknown. In retrospect, we speculate that the long-term intravenous mannitol treatment may have been a cause.

PRES and ATL have been described by McKinney et al., and have overlapping etiologies [5, 7]. The etiologies of RESLES may overlap with PRES and ATL, it also likely involves a component of endothelial injury and perhaps toxic demyelination [8, 9], which has been suggested as both a cause of PRES and ATL, based on histopathology. They also envisioned that RESLES is a variant of ATL (or a subtype), and endothelial injury may be a reason for the concomitant PRES- RESLES(MRES). As Mannitol can reduce brain edema, it may have ameliorated the injury, although in higher amounts it has been described to induce endothelial injury as well. However, we feel it is still imperfection in using the mechanism of endothelial injury alone to explain the case.

Previous studies of RESLES evaluated by diffusion tensor imaging (DTI) showed that the fractional anisotropy value for the lesion in SCC did not exhibit a significant decrease; this suggested that the lesions are located inside or beneath myelin [10]. However, Dan et al. reported cases of neonatal RESLES [11]; this contradicts the myelin sheath theory because neonates exhibited the disease even though their myelin sheaths are not yet developed. Accordingly, we speculate that the pathological changes seen in RESLES actually occur in astrocytes and are not associated with myelin sheaths [12]. This is also suggested by the study of Starkey et al. [13]. Aquaporins (AQPs) are an evolutionary conserved family of membrane transporter proteins that regulate the flow of water and, in some cases, glycerol and other small molecules across cellular membranes [14]. AQP4 is the most abundant water channel found in all brain structures in contact with the cerebral vascular compartment, participating in the water balance of the central nervous system [15]. A high concentration of AQP4 is found in the end-feet of astrocytes in contact with all blood vessels, and the AQP4 distribution in astrocytes differs significantly within brain structures such as the hippocampus, brainstem, and, in particular, corpus callosum [14, 15]. Early hypothesis suggests that severe hypertension exceeds the autoregulatory limits of the cerebral vasculature and leads to breakthrough of the blood–brain barrier, fluid leakage, and vasogenic edema. Endothelial injury is now considered a more common pathway of injury for most PRES patients [8]. Animal experiments showed that hyperosmotic stress induced by mannitol solution increased AQP4 expression in cultured rat astrocytes [16]. Because AQP4 expression in astrocytes was shown to be induced by hyperosmotic mannitol solution, which is commonly used to reduce brain edema, it has been suggested that AQP4 plays an important role in the treatment of brain edema. Interestingly, many previous studies [17, 18] have also suggested a dual role for AQP4 in the edema process: deleterious during cytotoxic edema formation and beneficial during the angiogenic edema resolution phase. On the basis of all these findings, we believe that the vasogenic edema in PRES was reduced with mannitol treatment, which increased the hyperosmotic stress and opened the blood–brain barrier; meanwhile, upregulation of AQP4 expression secondary to the increased osmotic pressure resulted in cytotoxic edema in the astrocytes in SCC (RESLES). In addition, the upregulation of AQP4 expression may have contributed to the regression of vasogenic edema in PRES. In most cases, the clinical and imaging abnormalities of RESLES are reversible. However, in some cases (such as in patients with severe hypoglycemia), if it is not treated in time, marked central nervous system damage may occur (can be seen permanently on T2 weighted imagines). Based on our hypothesis about the mechanism of RESLES, the upregulation of AQP4 is temporary and slight in most cases, so the clinical and imaging manifestations of most patients are reversible after the etiology is controlled or proper treated. To the best of our knowledge, this pathogenesis for RESLES has never been proposed.

## Conclusion

The sequential occurrence of eclampsia-associated PRES and RESLES in our patient prompted us to propose a novel pathogenesis for RESLES. Specifically, we speculate that AQP4 may be a key factor in the pathogenesis of the cytotoxic edema observed in the astrocytes in SCC. However, further large-scale studies are necessary to confirm this theory.

## Abbreviations

ADC: Apparent diffusion coefficient
AQP: Aquaporin
DWI: Diffusion-weighted imaging
FLAIR: Fluid-attenuated inversion recovery
MRI: Magnetic resonance imaging
PRES: Posterior reversible encephalopathy syndrome
RESLES: Reversible splenial lesion syndrome
SCC: Splenium of the corpus callosum
